# 1481. Clinical profile of cerebral small vessel disease in people with HIV hospitalized for ischemic stroke

**DOI:** 10.1093/ofid/ofad500.1317

**Published:** 2023-11-27

**Authors:** Folusakin Ayoade, Salma Hernandez

**Affiliations:** University of Miami, Miami, Florida; University of Miami Miller School of Medicine, Miami, Florida

## Abstract

**Background:**

The risk of ischemic stroke (IS) is twice as likely in people with HIV (PWH) than in the normal population. Cerebral small vessel disease (CSVD), a well-recognized risk factor for IS is poorly studied in PWH and represents a missed opportunity for targeted surveillance and risk mitigation for IS in this population.

**Methods:**

We retrospectively analyzed brain imaging (Computerized tomography and magnetic resonance imaging) findings of PWH obtained up to 8 years prior to the time of current admission for IS at our health institution. IS admission was between January 1, 2008 and December 31, 2018. We extracted information that suggests CSVD either in the imaging description or report summary. The terminologies include "ischemic leukomalacia", "white matter hyperintensities", "age related white matter changes", "lacunar infarcts" and "microbleeds". We analyzed demographic and clinical information including co-morbidities, HIV data, stroke severity data, hospital course and outcome. Several parameters of interest were analyzed in those with CSVD on brain imaging versus those without.

**Results:**

Out of the 70 HIV positive patients analyzed who presented with IS during the time period, 54 or 77% had CSVD on a previous brain scan within the prior 8 years preceding the current stroke. Other demographic and clinical information are illustrated in Table 1.

Only 66% of the cohort had a Glasgow coma scale (GCS) of 15 at admission. The NIH stroke scale of 5 or greater was 50%. Individuals without CSVD were younger (Mean 52.8 ± 9.5) compared to those with CSVD (Mean 60.1 ± 9.3), p=0.01. CSVD was correlated to age and CD4 counts with Pearson correlation of 0.319, p=0.007 (age) and 0.364, p=0.012 (CD4) respectively. There was no difference in stroke severity scores, ICU and hospital length of stay, and outcome in both groups.

**Table 1:**
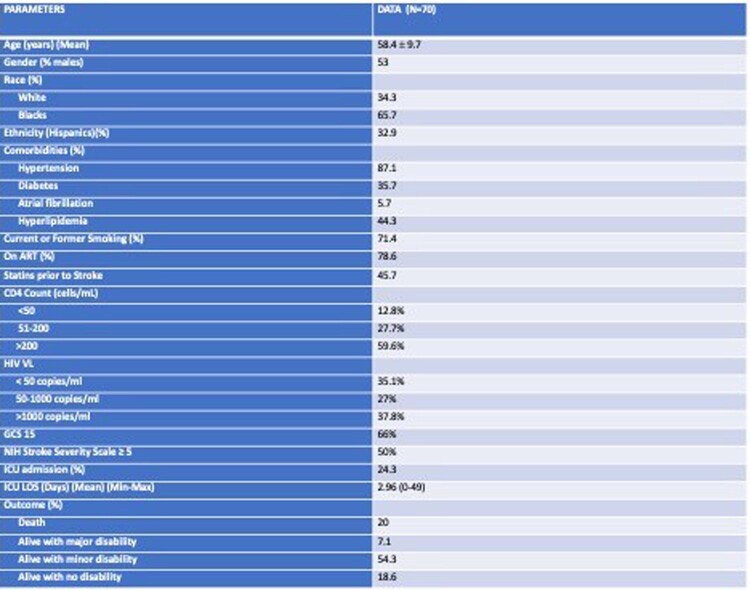
Demographic and Clinical information of CSVD in PWH (N=70). Legend: ART: Antiretroviral therapy VL: Viral load GCS: Glasgow Coma scale ICU: Intensive care unit LOS: Length of stay Min: Minimum value Max: Maximum value

**Conclusion:**

In this descriptive analysis of PWH presenting for hospital admission due to ischemic stroke, 77% had CSVD on a previous brain scan. Mortality was 20% with over 60% surviving with disabilities. CSVD in PWH is correlated to age and CD4 count. Further studies are needed to understand the interaction between CSVD and other host specific mitigating factors including demographics, co-morbidities, HIV factors and microbiome and their roles in contributing to excess stroke risk in PWH.

**Disclosures:**

**All Authors**: No reported disclosures

